# Ecology of food waste chain-elongating microbiome

**DOI:** 10.3389/fbioe.2023.1157243

**Published:** 2023-04-11

**Authors:** Simona Crognale, Alessio Massimi, Michela Sbicego, Camilla Maria Braguglia, Agata Gallipoli, Giulio Gazzola, Andrea Gianico, Barbara Tonanzi, Francesca Di Pippo, Simona Rossetti

**Affiliations:** Water Research Institute, National Research Council of Italy, CNR-IRSA—Istituto di ricerca sulle acque, Roma, Italy

**Keywords:** microbiome, microbial chain elongation, biotic interactions, multivariate statistics, DNA/RNA sequencing functional prediction, medium chain fatty acids, caproate, food waste

## Abstract

Microbial chain elongation has emerged as a valuable bioprocess for obtaining marketable products, such as medium chain fatty acids usable in several industrial applications, from organic waste. The understanding of the microbiology and microbial ecology in these systems is crucial to apply these microbiomes in reliable production processes controlling microbial pathways to promote favourable metabolic processes, which will in turn increase product specificity and yields. In this research, the dynamics, cooperation/competition and potentialities of bacterial communities involved in the long-term lactate-based chain elongation process from food waste extract were evaluated under different operating conditions by DNA/RNA amplicon sequencing and functional profile prediction. The feeding strategies and the applied organic loading rates strongly affected the microbial community composition. The use of food waste extract promoted the selection of primary fermenters (i.e., *Olsenella*, *Lactobacillus*) responsible for the *in situ* production of electron donors (i.e., lactate). The discontinuous feeding and the organic loading rate 15 gCOD L^-1^ d^-1^ selected the best performing microbiome in which microbes coexist and cooperate to complete the chain elongation process. Both at DNA and RNA level, this microbiome was composed by the lactate producer *Olsenella*, the short chain fatty acids producers *Anaerostipes, Clostridium sensu stricto 7, C. sensu stricto 12, Corynebacterium,* Erysipelotrichaceae *UCG-004, F0332, Leuconostoc*, and the chain elongator *Caproiciproducens*. This microbiome also showed the highest predicted abundance of short-chain acyl-CoA dehydrogenase, the functional enzyme responsible for the chain elongation process. The combined approach herein used allowed to study the microbial ecology of chain elongation process from food waste by identifying the main functional groups, establishing the presence of potential biotic interactions within the microbiomes, and predicting metabolic potentialities. This study provided pivotal indications for the selection of high-performance microbiome involved in caproate production from food waste that can serve as a basis for further improving system performance and engineering the process scale-up.

## Introduction

The usage of organic waste as new raw materials for the production of high-added value products is a central challenge of circular economy strategy aimed at waste valorisation and environmental impact minimization (Agler et al., 2011; Atasoy et al., 2018; Wu et al., 2019). Among the various bioproducts, the medium chain fatty acids (MCFAs) have recently garnered much interest due to their high market value and the wide range of industrial applications as precursors of liquid biofuels or commercial chemicals (Han et al., 2019; Wang and Yin, 2022). MCFAs are produced by chain elongation (CE) (Wu et al., 2021; Wang and Yin, 2022), an anaerobic microbial process in which short chain fatty acids (SCFAs) are converted to MCFAs by using mainly ethanol or lactate as electron donor (ED) (Angenent et al., 2016). The organic waste, rich in sugars, are valuable substrates for CE process promoting the primary fermentation for ethanol or lactate generation (Tang et al., 2016; Darwin et al., 2018), thus avoiding the addition of external EDs for the process. With this purpose, food waste (FW) can be used as substrate in this bioprocess allowing the *in situ* generation of EDs (mainly lactate) and electron acceptors (EAs) required for the MCFAs production (Wang et al., 2020). Although the composition of FW can widely vary depending on different sources, caproate (C_6_) was always revealed as the main MCFA produced (Wang and Yin, 2022). The biological production of caproate and others MCFAs from organic waste requires different steps, mediated by various microorganisms (Zhu et al., 2021; Kim et al., 2022b). Firstly, in the absence of external additions, lactate and ethanol are produced by primary fermentative bacteria (e.g., *Lactobacillus*, *Olsenella*, *Streptococcus*, and various members of the Prevotellaceae family) (Duber et al., 2018; Scarborough et al., 2018; Contreras-Dávila et al., 2020). These EDs are used for the production of SCFAs in a secondary fermentation step mediated by microorganisms mainly belonging to families Clostridiaceae, Eubacteriaceae, and Lachnospiraceae within phylum *Firmicutes* (Agler et al., 2012; Nzeteu et al., 2018; Shrestha et al., 2022). Lastly, SCFAs are elongated to MCFAs through the reverse β-oxidation (RBO) pathway mediated by chain elongating bacteria as well as *Caproicibacterium lactatifermentans*, *Caproiciproducens* sp. 7D4C2, *Clostridium kluyveri*, *Megaphaera elsdenii, Megasphaera hexanoica, Pseudoramibacter alactoliticus*, and Ruminococcaceae bacterium CPB6 (Tao et al., 2017; Reddy and Mikrobiologie, 2018; Contreras-Dávila et al., 2020; Candry and Ganigué, 2021; De Groof et al., 2021; Esquivel-Elizondo et al., 2021; Wang et al., 2022). Given the importance of microorganisms in the CE process, the understanding of the microbiology and microbial ecology in these systems is crucial to apply these microbiomes in reliable production processes and to optimise bioprocess performance increasing product specificity and yields.

Recently, the stability and feasibility of long-term conversion of FW to caproate in a single-stage system without the addition of external EDs were investigated (Crognale et al., 2021; Gazzola et al., 2022). Although the taxonomic composition of the microbial communities involved in the CE process has been previously described, the ecological mechanism for the biological production of high concentrations of caproate *via* this lactate-based CE process has yet to be fully elucidated. Indeed, engineered microbial ecosystems can be valuable environments for studying microbial ecology phenomena (Briones and Raskin, 2003; Daims et al., 2006; Griffin and Wells, 2017). The deepest understanding of the microbial ecology, including the complex interplay between biotic interactions and operation conditions, of CE process is a key-point for the performance improvement and engineering of the system up to large-scale applications. With this focus, a combined approach based on DNA/RNA sequencing and functional profile prediction of different mixed microbial communities developed in single-stage reactors for the long-term conversion of FW extract to caproate has herein been applied. In detail, this work is aimed at: i. identifying microbial functional groups involved in primary fermentation and CE process and evaluating their selection under various operating conditions (e.g., feeding strategy, organic loading rate); ii. identifying potential biotic factors (e.g., cooperation and/or competition) driving the diversity within the microbiome; iii. evaluating the activity of microbial functional groups by analysing total (DNA) and active (RNA) communities; iv. predicting metabolic potentialities of microbiomes involved in the primary fermentation and CE process.

## Materials and methods

### Reactor configuration and operation parameters

The investigation of acidogenic fermentation and CE processes was performed by sequentially operating various mesophilic fermenters with a working volume of 3 L fed with the liquid extract of FW, as previously described (Crognale et al., 2021; Gazzola et al., 2022). FW was collected from the cafeteria of the research area “Roma 1” of the National Research Council and consisted of mixed raw and cooked food such as cheese (15%), bread and pasta (15%), fruit and vegetable peelings (70%). FW was collected in multiple acquisitions and was manually screened in order to maintain such fixed composition typical of household FW. Successively, sorted scraps were firstly manually chopped and then shredded (particle size below 1 cm) by a lab-scale knife mill, prior to being stored at −20°C. A stock of raw FW was centrifuged via a lab-scale centrifuge Rotanta 460 (Hettich, Germany) operating at 4600 rpm for 10 min to obtain the extract to be fed to the reactors.

For the start-up, each fermenter was seeded with a different inoculum (depending on the period of operation) deriving from digested sludge collected at a municipal wastewater treatment plant, spiked with biomass coming from lab-scale anaerobic reactors treating FW. The six different operating conditions considered in this work are summarized in Table 1. In particular, a daily feeding strategy (every day from Monday to Thursday, defined “continuous” throughout the text) was used for the reactors #1 and #2. Otherwise, a “discontinuous” feeding strategy consisting in two feeding days per week (Monday and Thursday) was applied for the reactors #3, #4, #5, and #6. The applied organic loading rate (OLR) was in the range between 5 and 20 gCOD L^-1^d^-1^ (Table 1). In all the tests, hydraulic retention time (HRT) and temperature were kept constant at 4 days and 37°C ± 2°C, respectively. As reported in Table 1, further details regarding the operating conditions adopted during the experimental tests are reported in Crognale et al. (2021) and Gazzola et al. (2022).

**TABLE 1:** Overview of the reactors considered in this work and the relative experimental conditions.

Reactor #	Feeding strategy	OLR (gCOD L^-1^d^-1^)	References
1	Continuous	5	Crognale et al. (2021)
2	Continuous	15	Crognale et al. (2021)
3	Discontinuous	15	unpublished^a^
4	Discontinuous	15	Gazzola et al. (2022)
5	Discontinuous	20	Gazzola et al. (2022)
6	Discontinuous	20	unpublished^a^

^a^ The operating conditions adopted in the (unpublished) Test#3 and Test#6 are the same as in the Test#4 and Test#5, respectively.

The pH in all reactors was controlled every day and adjusted at 6 ± 0.2 by addition of 2.7 M of Na_2_CO_3_ or 4.5 M HCl when needed. S- and MCFAs concentrations, from acetic to caproic, were analysed by injecting 1 µL of filtered (0.22 µm porosity) liquid sample into a Perkin Elmer Auto System gas-chromatograph equipped with a FID detector (flame ionisation detector).

### Sample collection and DNA/RNA extraction

A total of 37 anaerobic sludge samples were weekly collected over the long-term operation of the six different reactors (totally lasted around 3 years). Small aliquots (2 mL) were immediately centrifuged at 15,000 rpm for 2 min; the obtained pellet was stored at −20°C until DNA extraction performed with a DNeasy PowerSoil Pro Kit (QIAGEN, Italy). The extracted DNA was eluted in 100 μL of nuclease-free water. At the end of operation, during the last sampling day of each reactor the pellet obtained from two additional small aliquots (2 mL) of anaerobic sludge was immediately stored at −80°C by adding RNAprotect^®^ Bacteria Reagent (QIAGEN, Italy) to prevent RNA degradation. The RNeasy PowerSoil Total RNA Kit (QIAGEN, Italy) was used for RNA extraction according to the manufacturer’s instructions. The extracted RNA was eluted in 50 μL of RNase-free water and subsequently converted to cDNA using the iScript Select cDNA Synthesis kit (BIO-RAD, United States). The final quality and quantity of DNA and cDNA was validated by 1% agarose gel electrophoresis, Nanodrop 3300 (ThermoScientific, Monza, Italy), and Qubit 3.0 Fluorometer (Thermo Fisher Scientific, Waltham, MA, United States).

### 16S rRNA amplicon sequencing and data processing

The DNA and cDNA extracts were used as template for the high-throughput sequencing of the 16S rRNA gene following library preparation and protocol reported in (Crognale et al., 2019). In detail, the primer pair 27F (5′-AGA​GTT​TGA​TCC​TGG​CTC​AG-3′) and 534R (5′-ATTACCGCGGCTGC TGG-3′) was used for the amplification of V1-V3 region of bacterial 16S rRNA gene. Each PCR reactions was set up in 25 μL volumes containing 15 ng of DNA/cDNA, 0.5 μM primers and 1 × Phusion Flash High-Fidelity PCR Master Mix (Thermo Fisher Scientific, United States). The PCR settings consisted in an initial denaturation at 98°C for 10 s, 30 cycles of 98°C for 1 s, 60°C for 5 s, 72°C for 15 s and final elongation at 72°C for 1 min. The Agencourt^®^ AMpure XP bead protocol (Beckmann Coulter, United States) was applied for the purification of amplicon libraries. Subsequently, 5 μL of amplicon library was used as template in a second PCR reaction (50 μL volume) containing 1× Phusion Flash High-Fidelity PCR Master Mix (Thermo Fisher Scientific, United States) and Nextera XT Index Primers (Kit v2, Set A—Illumina, United States). The PCR settings consisted in an initial denaturation at 98°C for 10 s, 8 cycles of 98°C for 1 s, 55°C for 5 s, 72°C for 15 s and final elongation at 72°C for 1 min. Also in this case, the purification of amplicons was performed by applying the Agencourt^®^ AMpure XP bead protocol (Beckmann Coulter, United States). The Qubit 3.0 Fluorometer (Thermo Fisher Scientific, United States) was used for the quantification of library concentration. The purified libraries were pooled in equimolar concentrations and diluted to 4 nM. The samples were paired end sequenced (2 bp × 301 bp) on a MiSeq platform (Illumina, United States of America) using a MiSeq Reagent kit v3, 600 cycles (Illumina, United States of America) following the standard guidelines for preparing and loading samples, with 20% Phix control library.

The raw sequences were quality checked by using fastqc and then processed and analysed using QIIME2 v. 2018.2 (Bolyen et al., 2019). The demux (https://github.com/qiime2/q2-demux 10/02/2018) and cutadapt (https://github.com/qiime2/q2-cutadapt 02/12/2017) plugins were used in order to demultiplex reads and to remove primer sequences. The obtained reads were denoised, dereplicated and chimera-filtered using DADA2 pipeline and amplicon sequence variants (ASVs) were identified (Callahan et al., 2016; 2017). The reads were subsampled and rarefied at the same number for each sample by using the feature-table rarefy plugin (Weiss et al., 2017). The taxonomy was assigned to ASVs using a pre-trained naïve-bayes classifier based on the 16S rRNA at a 99% similarity of the SILVA132 release (Quast et al., 2013).

The already published datasets obtained from the DNA sequencing of reactors #1, #2, #4, and #5 are available through the Sequence Read Archive under accession numbers PRJNA872912 (DNA sequencing for reactors #1 and #2) and PRJNA809915 (DNA sequencing for reactors #4 and #5). The not yet published datasets comprising DNA sequences of reactors #3 and #6 and the cDNA of all reactors presented in this work are available under accession number PRJNA872917.

Due to the different origin of inocula, the sequencing data obtained from the samples taken at the beginning of the operation of each reactor were not considered in the statistical elaboration and functional profile prediction described below.

### Statistical analysis

Biodiversity indices (Dominance, Simpson, Evenness, Shannon, Margalef, Chao-1) were calculated for each reactor.

Similarity matrices of bacterial community composition under continuous and discontinuous feeding strategies were calculated using sequencing data and applying the relative abundance-based Bray-Curtis index. A non-metric Multi-Dimensional Scaling ordination plot (nMDS) was used to visualize the variation patterns of the main genera (≥1% of total reads). The values of relative abundance were incorporated into the nMDS analysis with a vector-fitting procedure, in which the length of the arrow is proportional to the contribution of each variable to the nMDS-axes.

In order to assess the effect of the applied OLR the principal component analysis (PCA), based on the correlation matrix, was performed by separately comprising the relative abundances of the microbial taxa in the reactors operated under continuous and discontinuous feeding conditions. According to the relative abundance criterion (Risely, 2020; Neu et al., 2021), only genera ≥5% of total reads were considered as microbial core taxa.

The non-parametric multivariate analysis of variance (PERMANOVA) was used to test the difference in microbial community composition between reactors operated under different feeding strategies (continuous vs discontinuous) and applied OLRs (5 gCOD L^-1^d^-1^ vs. 15 gCOD L^-1^d^-1^, and 15 gCOD L^-1^d^-1^ vs. 20 gCOD L^-1^d^-1^).

The non-parametric Kruskal-Wallis univariate test was performed by comprising the microbial genera mainly responsible for the clustering observed in the PCA biplots (significantly correlated with *x*-axis) in order to assess statistical differences between the sample groups.

The Principal Coordinate Analysis (PCoA) ordination plot was used to visualize the relatively closer associations among the lactate-producing, fermentative and chain-elongating core taxa (genera ≥5% in at least one sample), thus classified according to the bacterial genera description available in literature.

Correlations between the microbiota, S- and MCFAs, ethanol, and lactate were identified using Spearman’s correlation.

The differences between DNA and RNA community profiles were evaluated using analysis of variance (ANOVA).

Statistical analyses were performed by using PAST software package (Palaeontological STatistics, ver. 4.04) (Hammer et al., 2001).

### Functional profile prediction

The metagenomics and metatranscriptomics potential of microbial communities selected in the bioreactors under different operating conditions was predicted using 16S rRNA sequencing data (ASVs representative sequences and abundance) via PICRUSt2 (https://github.com/picrust/picrust2) with default parameters (Douglas et al., 2020). Functional gene prediction was obtained from Kyoto Encyclopaedia of Genes and Genomes-Orthology (KO). Information on metabolic pathways and Enzyme Commission (EC) numbers involved in the production of EDs (i.e., ethanol, lactate) and the RBO pathway were manually categorized based on the KEGG databases.

## Results

### Fermentation and chain elongation of FW extract under different operating conditions

During fermentation tests, S-and MCFAs composition and concentration strongly varied in the reactors operated under different operating conditions (Supplementary Tabel S1, Supplementary Figure S1). The maximum caproate production (up to ~ 7 g L^-1^) was observed in the reactor #3 and #4 operated at OLR 15 gCOD L^-1^d^-1^ with a discontinuous feeding strategy. Furthermore, in these two reactors the highest proportion of caproate (up to 21.5%) compared to the total fatty acids was retrieved. On the contrary, the lowest caproate production (as % of total fatty acids) was obtained in reactor #1 (~ 8%) operated at OLR 5 gCOD L^-1^d^-1^ with a continuous feeding and reactors #5 and #6 (~ 15%) operated at OLR 20 gCOD L^-1^d^-1^ with a discontinuous feeding. As a general trend in all reactors, SCFAs were mainly produced during first feeding cycles and the CE took place roughly starting from the third/fourth feeding cycle. Furthermore, lactate was *in situ* produced in all reactors immediately after each feeding and then quickly consumed; at the same time low and constant ethanol concentrations were retrieved (Crognale et al., 2021; Gazzola et al., 2022).

### Composition and diversity in the total (DNA) microbial community

The high-throughput sequencing of the 16S rRNA gene based on DNA extracts revealed different microbial communities’ composition in the reactors operated under different experimental conditions (Supplementary Figure S2). In particular, the reactor #1 (OLR 5 gCOD L^-1^d^-1^ and continuous feeding) was mainly inhabited by genera *Streptococcus* (on average 56% of total reads), *Succiniclasticum* (9.7%), *Pyramidobacter* (7.8%), and *Acidaminococcus* (7.4%). Differently, the genera *Olsenella* (30.8%), *Actinomyces* (22.7%), *Sutterella* (15.2%), *Pseudoramibacter* (11.6%), and *Lactobacillus* (3.2%) characterized the microbiome selected by the operation conditions of reactor #2 (OLR 15 gCOD L^-1^d^-1^ and continuous feeding). The reactors #3 and #4 (OLR 15 gCOD L^-1^d^-1^ and discontinuous feeding) hosted bacterial communities mostly composed by genera *Olsenella* (up to 87.3%), *Catenisphaera* (up to 78.2%), *Caproiciproducens* (up to 50.7%), *Corynebacterium* (up to 40.5%), *Clostridium sensu stricto* 7 (up to 38.9%), and *C. sensu stricto* 12 (up to 17.2%). Lastly, the microbial communities developed in reactors #5 and #6 (OLR 20 gCOD L^-1^d^-1^ and discontinuous feeding) mainly comprised *Olsenella* genus (up to 99.0%), followed at minor extent by *Lactobacillus* (up to 53.7%), *Caproiciproducens* (up to 4.8%), and *C. sensu stricto* 12 (up to 4.2%).

Overall, microbial communities inhabiting reactors operated at OLR 20 gCOD L^-1^d^-1^ showed a very low biodiversity at single ASV level (0.5 < D < 0.9) with an outlier value represented by d11 of Reactor #5 (D = 0.1) (Supplementary Figure S3), as also confirmed by Simpson and Evenness indices. The microbial communities selected in the reactors operated at OLR 15 gCOD L^-1^d^-1^ showed the highest species richness as indicated by Shannon, Margalef, and Chao-1 indices.

### Effect of the feeding strategy on the microbial community composition

**FIGURE 1:**
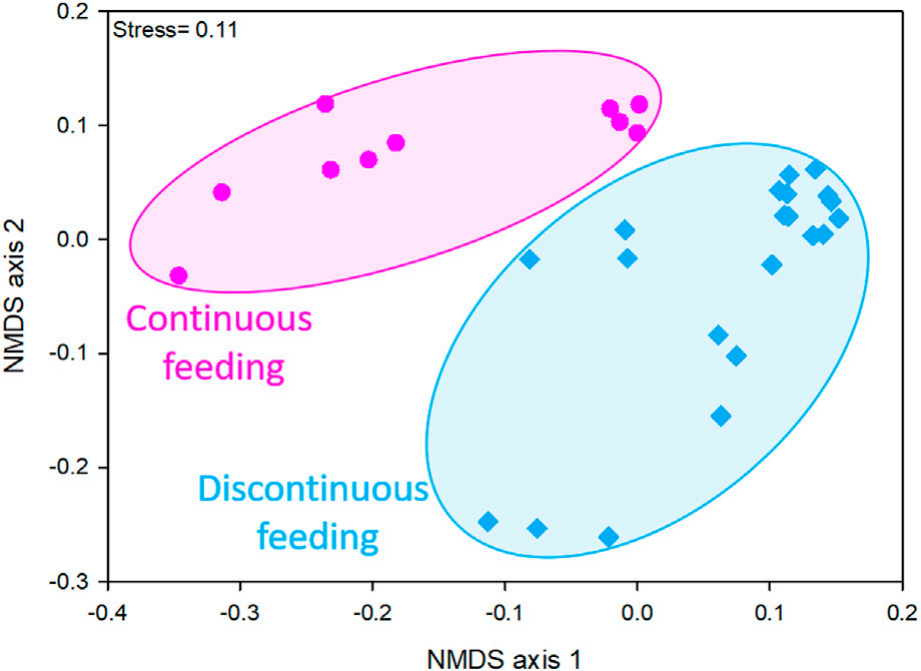
NMDS ordination plots, based on Bray-Curtis distance matrixes of log (X+1)-transformed sequencing data (community at genus level ≥1% in at least one sample). The stress value (i.e., <0.2) suggests for an accurate representation of the dissimilarity among samples.

Taxon composition of microbial communities varied depending on the different feeding conditions imposed to the reactors, as showed by the NMDS ordination plot (Figure 1). In fact, considering data obtained by the sequencing of 16S rRNA gene on DNA extracts, two distinct clusters were evident, one resembling microbiomes of discontinuously fed reactors and one of the continuously fed reactors communities. This evidence was confirmed by the significant differences retrieved between the two clusters by PERMANOVA analysis (Bray-Curtis *p* < 0.01).

### Effect of the OLR on the microbial community composition

**FIGURE 2:**
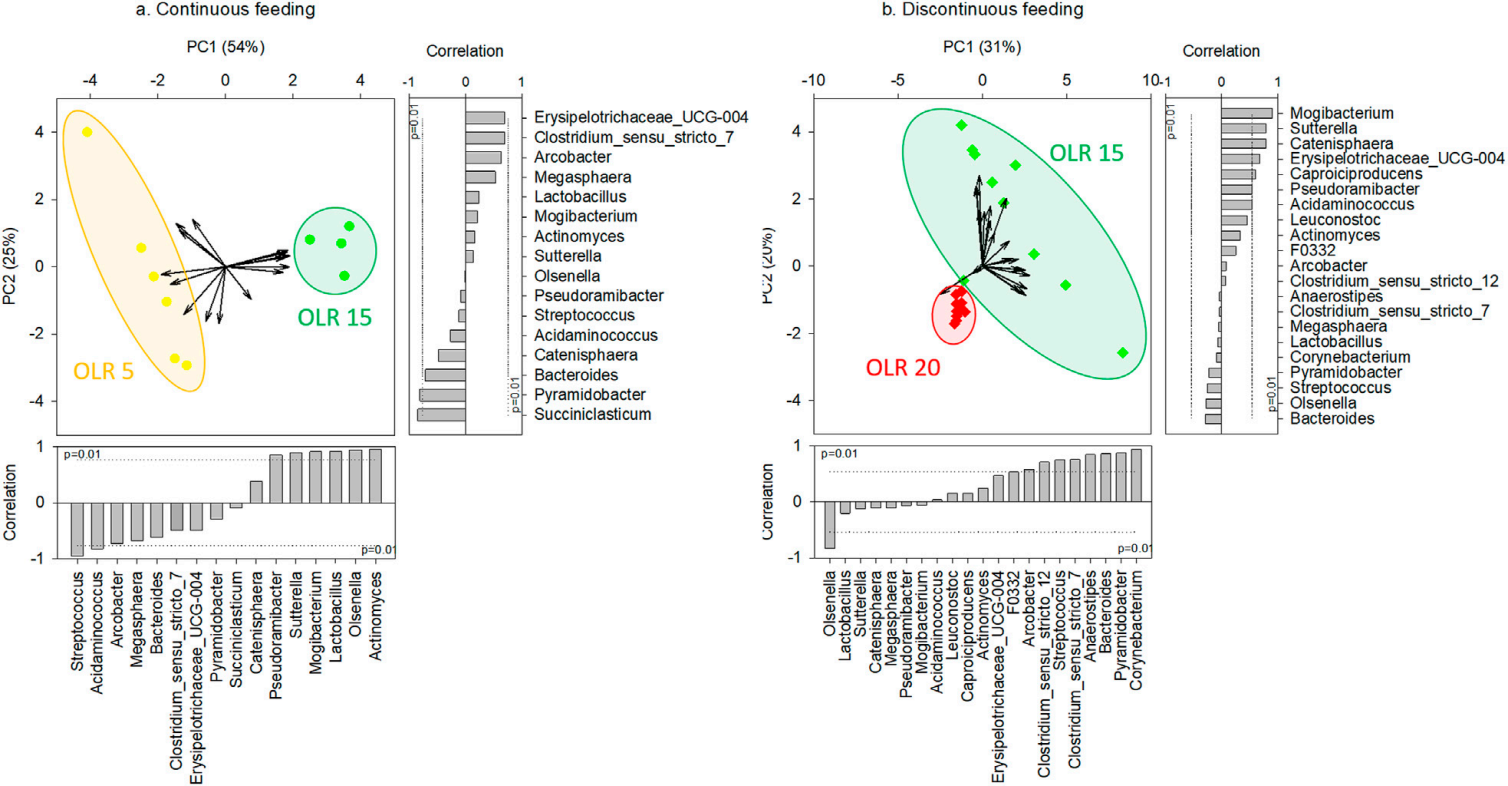
Principal Components Analysis (PCA) biplot representing the typifying microbial composition at genus level (≥5% in at least one sample) in reactors operated at different OLRs under continuous **(A)** (reactors #1 and #2) and discontinuous **(B)** feeding strategies (reactors #3, #4, #5, and #6). The vector length is proportional to the correlation between corresponding parameter and the PCA axis 1 and 2. Bar plot shows the contribution of each variable (vector projection values) expressed as the correlation with the x- and *y*-axis. The genera significantly correlated with x- and *y*-axis were the main responsible for the obtained clusterization.

Considering the same feeding strategy, the applied OLR strongly affected the microbial community composition in the reactors. In particular, regarding continuous feeding, significant differences were obtained between genus composition of communities grown in the reactors operated at OLR 5 gCOD L^-1^d^-1^ and at OLR 15 gCOD L^-1^d^-1^, as highlighted by PCA analysis (Figure 2A) and confirmed by PERMANOVA test (*p* < 0.01). Furthermore, significant differences (Kruskal-Wallis test, *p* < 0.05) were observed between the genera correlated with *x*-axis in the PCA and most responsible for the obtained grouping (i.e., *Streptococcus*, *Acidaminococcus*, *Pseudoramibacter*, *Sutterella*, *Mogibacterium*, *Lactobacillus*, *Olsenella*, *Actinomyces*).

Likewise, considering discontinuous feeding, the effect of OLR was observed on the microbiome composition. In particular, as highlighted by PCA analysis (Figure 2B) and confirmed by PERMANOVA test (*p* < 0.01), the microbiomes selected in the reactors operated at 15 gCOD L^-1^d^-1^ were significant different from those observed in the reactors at 20 gCOD L^-1^d^-1^. The latter also presented a low diversity, as shown by Alpha-diversity indices results (Supplementary Figure S3). The Kruskal-Wallis test highlighted significant differences (*p* < 0.05) between genera *Olsenella*, *Clostridium sensu stricto 12*, *Streptococcus*, *Clostridium sunsu stricto 7*, *Anaerostipes*, *Bacteroides*, *Pyramidobacter*, and *Corynebacterium*, significantly correlated with *x*-axis in the PCA and most responsible for the obtained clustering.

### Associations among the lactate-producing, fermentative and chain-elongating core taxa

**FIGURE 3:**
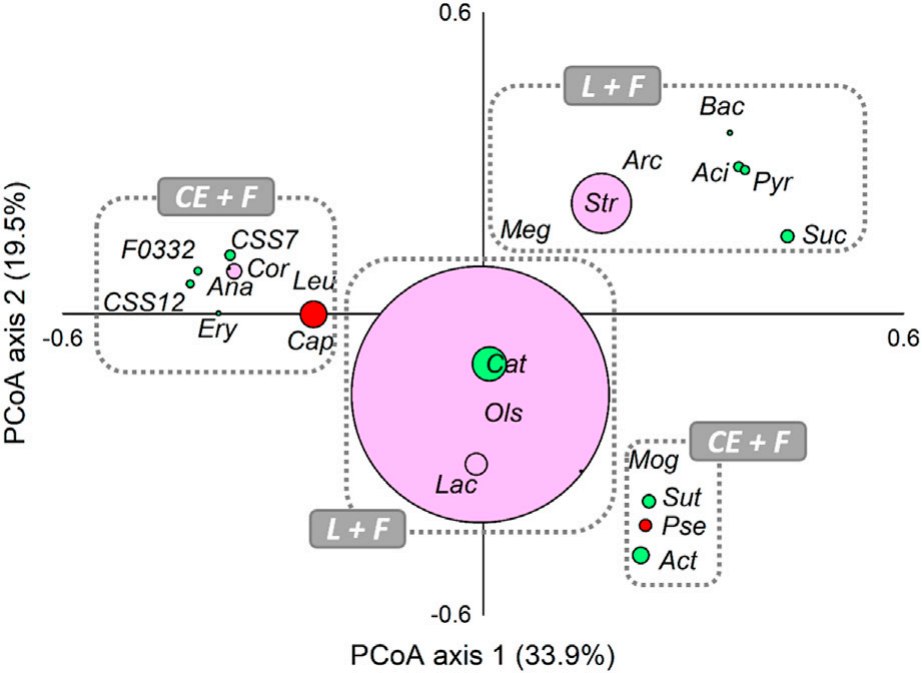
Principal Coordinate Analysis (PCoA), based on Bray-Curtis similarity index, of lactate-producing (L, pink), secondary fermentative (F, green), and chain-elongating (CE, red) core taxa at genus level (≥5% in at least one sample). The size of dots is proportional to the relative abundance (average %) of each core taxa. Taxa ordinated closer to one other showed more similar variation patterns than those ordinated further away. Aci, *Acidaminococcus*; Act, *Actinomyces*; Ana, *Anaerostipes*; Arc, *Arcobacter*; Bac, *Bacteroides*; Cap, *Caproiciproducens*; Cat, *Catenisphaera*; Cor, *Corynebacterium*; CSS7, *Clostridium sensu stricto* 7; CSS12, *Clostridium sensu stricto* 12; Ery, Erysipelotrichaceae UCG-004; F0332; Lac, *Lactobacillus*; Leu, *Leuconostoc*; Meg, *Megasphaera*; Mog, *Mogibacterium*; Ols, *Olsenella*; Pse, *Pseudoramibacter*; Pyr, *Pyramidobacter*; Str, *Streptococcus*; Suc, *Succiniclasticum*; Sut, *Sutterella*.

Moreover, PCoA analysis was computed on the core members (genera ≥5% in at least one sample) of all analysed communities for evaluating the presence of possible associations among lactate-producing, fermentative, and chain-elongating taxa in analysed reactors (Figure 3). Relatively closer associations were found between lactate-producing *Olsenella*, *Lactobacillus*, and the fermentative *Catenisphaera*. Notably, a closed association between fermentative *Sutterella*, *Mogibacterium*, and *Actinomyces* and chain-elongating *Pseudoramibacter* was also observed. *Capoiciproducens* showed an association with *Corynebacterium* and generally fermentative *Anaerostipes*, *Clostridium sensu stricto* 7 and *C. sensu stricto* 12, Erysipelotrichaceae UCG-004, F0332, and *Leuconostoc*.

**FIGURE 4:**
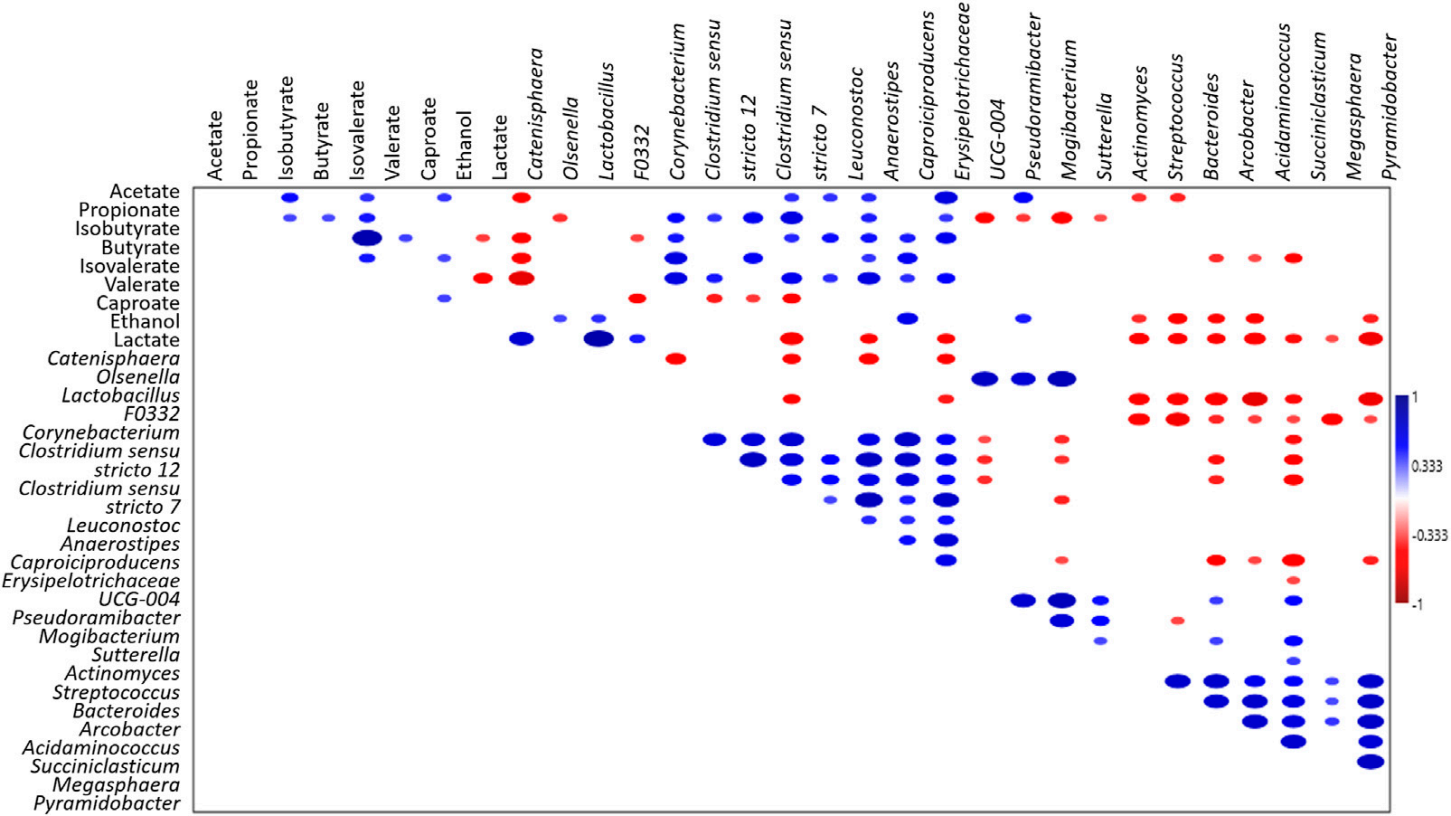
Spearman’s correlation analysis of microbiota (genera ≥5% in at least one sample),S- and MCFAs (mg L^-1^) and electron donors (mg L^-1^) in all the tested reactors. Blue and red ellipses indicated significant positive and negative correlation (*p* < 0.05), respectively. Colour saturation is proportional to the absolute correlation.

Notably, a positive correlation (*p* < 0.05, Figure 4) between the concentration of volatile fatty acids and the grouped genera *Anaerostipes*, *Capoiciproducens*, *Clostridium sensu stricto* 7, *C. sensu stricto* 12, *Corynebacterium*, Erysipelotrichaceae UCG-004, F0332, and *Leuconostoc* was retrieved by Spearman’s correlation analysis. On the contrary, a negative correlation (*p* < 0.05, Figure 4) was observed between the volatile fatty acids concentration and the grouped genera *Acidaminococcus*, *Arcobacter*, *Bacteroides*, *Megasphaera*, *Pyramidobacter, Streptococcus*, and *Succiniclasticum*. Moreover, positive correlation (*p* < 0.05) was obtained among caproate concentration and *Caproiciproducens*, *Catenisphaera*, *Mogibacterium,* and *Olsenella*, while negative correlation was retrieved with *Acidaminococcus*, *Arcobacter*, *Bacteroides*, *Pyramidobacter*, and *Streptococcus* (Figure 4).

### Composition and diversity in the active (RNA) microbial community

**FIGURE 5:**
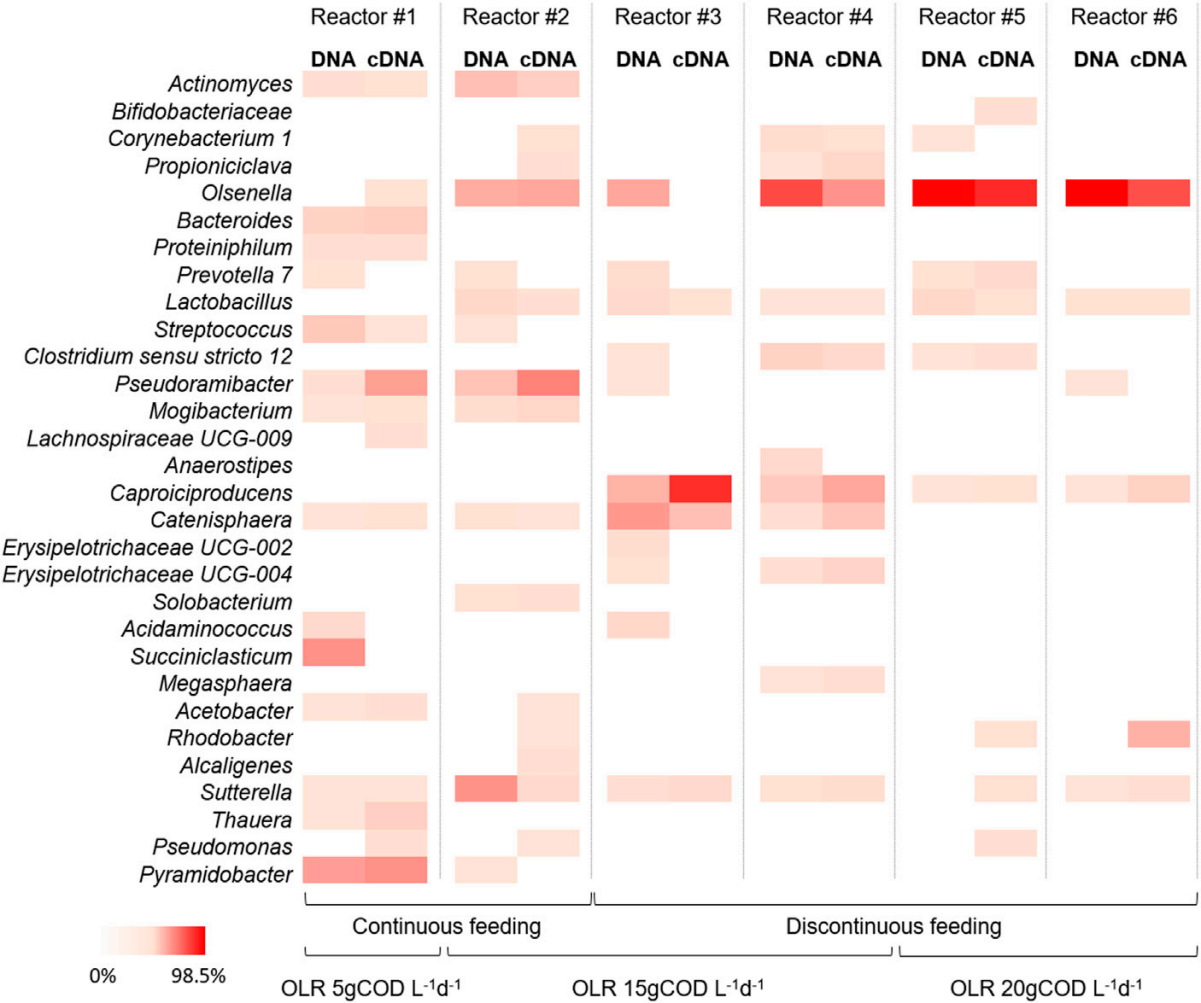
Heat-map representing the relative abundance of microbial genera (≥1% relative abundance of total reads in at least one sample) of the DNA and cDNA profiles of different samples taken during last operating day of the reactors at different OLRs and feeding strategies. The colour scale ranges from 0 (white) to 98.5% (red) relative abundance.

The 16S rRNA amplicon sequencing was also performed on RNA extracted from samples taken at the end of the operation of each reactor. The principal aim of the use of cDNA sequencing was to investigate the relations between active microorganisms and metabolic potentialities involved in chain elongation process. For this reason, the biomass for the cDNA analysis was sampled at the end of the tests, once the steady state condition was reached and in most cases the highest caproate concentration (of each test) was observed. The phyla *Actinobacteria* and *Firmicutes* represented the most active fractions of microbiomes encountering together between 66.7% and 94.7% of total reads. Among these phyla, no significant differences (*p* > 0.05) between total (DNA) and active (RNA) communities were observed in all reactors (Figure 5). In the continuously fed reactor #1 (OLR 5 gCOD L^-1^d^-1^) the active fraction of microbial community was mainly represented by genera *Pyramidobacter* (36.6% of total reads), *Pseudoramibacter* (29.6%), and *Bacteroides* (9.6%). In the reactor #2 (OLR 15 gCOD L^-1^d^-1^ and continuous feeding) the active microbiome was mainly composed by genera *Pseudoramibacter* (42.6%), *Olsenella* (26.5%), and *Actinomyces* (8.8%). The composition of the active microbiome in reactors #3 and #4 (OLR 15 gCOD L^-1^d^-1^ and discontinuous feeding) was mostly characterized by genera *Caproiciproducens* (up to 77.1%), *Olsenella* (up to 34.4%), *Catenisphaera* (up to 15.9%), Erysipelotrichaceae UCG-004 (up to 6.7%), and *Clostridium sensu stricto* 12 (up to 5.9%). Lastly, the active microbial communities observed in reactors #5 and #6 (OLR 20 gCOD L^-1^d^-1^ and discontinuous feeding) were mainly composed by genera *Olsenella* (up to 78.0%), followed at minor extent by *Caproiciproducens* (up to 7.5%), *Prevotella* 7 (up to 3.9%), and *C. sensu stricto* 12 (up to 1.9%).

### Functional prediction based on 16S rRNA sequencing data

**FIGURE 6:**
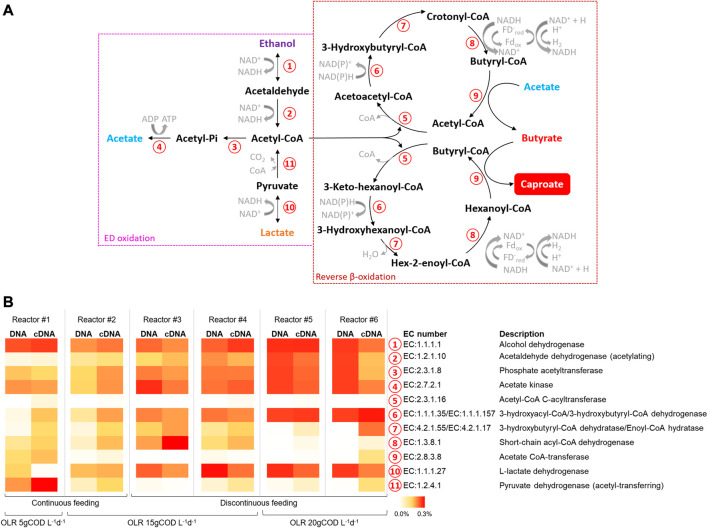
**(A)** Principal metabolic pathways in chain elongation process according to (Wu et al., 2019) **(B)** Relative abundances of predicted copy number of Enzyme Classification (EC) numbers related to chain elongation process (as % of total predicted EC numbers).

The metabolic potential of the microbiomes involved in CE process in all reactors, both at the DNA and RNA levels, was estimated considering the KEGG pathway database. In particular, the data elaboration was mainly focused on the enzymes involved in the oxidation/production of EDs and RBO, principal pathways in the CE process as graphically depicted in Figure 6A. The functional enzymes alcohol (EC1.1.1.1) and lactate (EC1.1.1.27) dehydrogenase, respectively related to the interconversion between ethanol and acetaldehyde and between lactate and pyruvate, showed the highest abundances (up to 0.48%) in reactors #5 and #6 (Figure 6B). The enzymes involved in the acetate formation from acetyl-CoA, namely, phosphate acetyltransferase (EC2.3.1.8) and acetate kinase (EC2.7.2.1), showed abundances ranging between 0.10% and 0.19% and 0.12% and 0.34% respectively in all reactors. All the functional enzymes involved in the RBO pathway (No. 5-9 in Figure 6A) were present in all reactors with differences depending on the applied operating conditions. Notably, the short-chain acyl-CoA dehydrogenase (EC1.3.8.1), key enzyme in the CE process, showed the highest abundance (up to 0.27%) in reactors #3 and #4.

## Discussion

The application of complex organic substrates has been widely demonstrated as an efficient strategy for the production of high-added value products such as MCFAs (Atasoy et al., 2018; Han et al., 2019; Lee et al., 2019; Stamatopoulou et al., 2020; Venkateswar Reddy et al., 2020; Albizzati et al., 2021). In this study and our previous works (Crognale et al., 2021; Gazzola et al., 2022), the use of the food waste extract, rich in bioavailable soluble sugars, has been revealed as an optimal strategy for promoting CE process, overriding biological hydrolysis and external ED addition widely proposed in other works (Grootscholten et al., 2014; Wang and Yin, 2018; Wang and Yin, 2022).

This study was focused on microbial ecology of microbiomes developed in engineered systems for caproate production with the aim to elucidate the function of diverse microorganisms and their biotic interactions, including cooperation and/or competition. Despite the restrictive environmental conditions experimented in these systems, microbes involved in the CE process may be indeed diverse and belonging to complex communities (Candry and Ganigué, 2021). Unravelling the functional microbial groups and evaluate their possible cooperation and/or competition also with other communities’ members will enable to contribute to design processes with better performances, by allowing the best operational conditions to favour the functionality of the key groups able to promote the CE process. This is particularly important when the bioprocess takes place in one-stage reactor, where the diverse functional microbial groups need to coexist and cooperate to complete the CE process (Candry and Ganigué, 2021).

The FW extracts herein used, strongly influenced the microbial community composition promoting the selection of primary fermenters (i.e., *Olsenella*, *Lactobacillus*) responsible for the *in situ* production of EDs (Kim et al., 2022b). In this work, the conditions (i.e., continuous/discontinuous feeding strategies, different OLRs) imposed to the reactors also positively selected secondary fermenters (i.e., *Caproiciproducens*, *Clostridium*, *Megasphaera*, Erysipelotrichaceae, *Pseudoramibacter*) contributing to S- and MCFAs production (Wang and Yin, 2022). Overall, the feeding strategies used in this work strongly affected the microbial community composition and diversity, as also discussed in previous reports (Kim et al., 2022a). In particular, a discontinuous feeding strategy seemed to exert a selective pressure that favoured highly functional and specialized microbial communities resulting in a strong selection of EDs-producing and chain elongating microorganisms, as especially observed in reactors #3 and #4. Conversely, continuous feeding conditions (i.e., reactors #1 and #2), selected communities not efficient in carrying out fermentation and CE. Most likely, in this case, the every-day purging of biomass and the limited time available for the utilization of all the substrate between two feeding cycles, favoured the differentiation of various ecological micro-niches, that did not allow for the selection of specialized and efficient EDs-producing and chain elongating microbiomes.

The OLR conditions herein tested strongly affected the microbial community composition, as also previously observed (De Groof et al., 2020; 2021; Mariën et al., 2022). The 15 gCOD L^-1^d^-1^ condition, indeed, selected microbiomes mainly composed by generally fermentative and chain-elongating microbes (i.e., *Caproiciproducens, Clostridium sensu stricto 7, C. sensu stricto 12, Corynebacterium,* Erysipelotrichaceae *UCG-004, Olsenella*). In contrast, in systems where higher OLR (20 gCOD L^-1^d^-1^) was experimented, lactate-producer microorganisms were selected at the expense of chain elongating bacteria. This was probably due also to the accumulation of caproate that reached the threshold of toxicity inhibiting the activity and selection of microorganisms (Ge et al., 2015; Allaart et al., 2021). Many researchers found that the production of n-caproate is hardly to exceed 12 g/L without *in-situ* product removal, even though the pH is higher than the pKa of the n-caproic acid (Steinbusch et al., 2011; Zhu et al., 2015; Roghair et al., 2018; Wu et al., 2021). The different caproate concentrations exhibiting toxicity depend strictly on the pH of the fermentation broth but also on the presence of other carboxylic acids. With a pKa of 4.85 for Caproic acid, 50% of the total carboxylate is found in the acidic form at a pH equal to the pKa. In this study, the pH dropped rapidly under 5 because of the rapid consistent formation of lactate requiring manual base dosage to maintain the pH around 6, suitable for CE process. The daily occasional manual pH control can hence determine the formation of toxic undissociated acids. During Test #5 and Test#6, in fact, the high concentration of caproate on the one hand, and the continuous bioavailability of fresh substrate on the other hand, might have inhibited the CE process resulting in a notable decrease of caproate-producing microorganisms. Such inhibition would also explain the simultaneous accumulation of lactate in the fermentation broth.

As extensively discussed in Gazzola et al. (2022), due to this inhibiting environmental condition, chain elongating bacteria cannot carry out normal metabolic activities, thus being progressively washed away from the system; after the stop-feeding, the system restored its capability to consume lactate and to complete the CE process, even if the lactate-producing microbiome was constantly present in the reactor.

The results obtained by data elaboration performed in this study allowed to identify common functional groups occurring in microbial communities and to describe their ecological dynamics within the systems. Three different functional microbial groups over the long-term operation of the reactors were identified: EDs-producers or primary fermenters (i.e., *Olsenella*, *Lactobacillus*), secondary fermenters responsible for the production of SCFAs (i.e., *Clostridium*, Erysipelotrichaceae)*,* and chain elongating microorganisms (*i.e., Caproiciproducens*, *Megasphaera*, *Pseudoramibacter*). Moreover, the close association found between microorganisms capable to produce S- and MCFAs, observed in those reactors (#3 and #4) where the highest production of fatty acids occurred, indicates that conditions experimented in these systems favored an efficient microbial cooperation. Furthermore, these two functional groups composed by genera *Anaerostipes*, *Caproiciproducens*, *C. sensu stricto* 7, *C. sensu stricto* 12, *Corynebacterium*, Erysipelotrichaceae UCG-004, F0332, and *Leuconostoc* were strongly correlated with fatty acids abundance, suggesting a direct or indirect contribution to caproate production. Therefore, the operating conditions imposed to reactors #3 and #4 (OLR 15 gCOD L^-1^d^-1^ and discontinuous feeding strategy) selected microbiomes where the different microbes coexist and efficiently cooperate for the substrate utilization promoting highest CE process performances.

The combined analysis of microbiomes based on the 16S rRNA gene and its transcripts has been performed to obtain a strong overview of dynamics in the total and active communities and to elucidate potential cooperation and competition between microorganisms and overall functionality (De Vrieze et al., 2018). In the present study, no significant differences between the total (DNA) and active (RNA) microbial communities have been found due to the high selection and activity of microorganisms in the reactors herein investigated. In fact, the occurrence of the three different functional microbial groups retrieved by sequencing of DNA amplicons was confirmed by the sequencing of RNA transcripts. In particular, overall, the highest abundant active genera were the lactate-producing *Olsenella*, the chain elongators *Caproiciproducens* and *Pseudoramibacter*, and various secondary fermentative microorganisms that guaranteed the formation of SCFAs. The similarity between DNA and RNA profiles strongly supported the comparative analysis of the metagenome and metatranscriptome based on metabolic pathways prediction. Recently, this predictive approach has been used on microbial communities involved in anaerobic bioprocess as, for example, dark fermentation, and CO_2_- and ethanol-based CE processes (Wenzel et al., 2018; Mugnai et al., 2021; Yin and Wang, 2021; Huo et al., 2022). This study provided, for the first time, new insights into the predictive metabolic pathways of mixed microbial communities grown in single-stage reactors involved in lactate-based CE process from FW. The co-existence of key enzymes involved in EDs oxidation/formation and RBO pathways was predicted in all reactors with differences depending on the applied operating conditions in agreement with process performance. These results are consistent with the metagenomics assessment previously performed on the reactor #2 where the presence of lactate dehydrogenase, pyruvate ferredoxin oxidoreductase, as well as the enzymes involved in RBO pathway suggested the occurrence of a lactate-based CE process (Crognale et al., 2021). In the present study, the short-chain acyl-CoA dehydrogenase, the functional enzyme responsible for CE of acetate to butyrate, of butyrate to caproate, and beyond, was mostly present in reactors #3 and #4, in line with the highest butyrate and caproate production. Notably, the enzymes involved in the EDs oxidation and subsequent production of acetyl-CoA and acetate were mainly present in reactors #5 and #6, in line with the highest OLR and lactate production.

These results further support what discussed above, confirming that the operative conditions experimented in reactors #3 and #4 (i.e., OLR 15 gCOD L^-1^d^-1^ and discontinuous feeding) selected communities where key microorganisms cooperate for an efficient utilization of substrates, thus resulting in high performance caproate production process.

## Conclusion

The combined approach herein used allowed to study the microbial ecology of CE process from FW extract by identifying the main functional groups, establishing the presence of potential microbial interactions within the microbiomes, and predicting metabolic potentialities. The discontinuous feeding and the OLR 15 gCOD L^-1^d^-1^ conditions resulted to select microbiomes composed by EDs producers (*Olsenella*), SCFAs-producers (*Anaerostipes*, *C. sensu stricto* 7, *C. sensu stricto* 12, *Corynebacterium*, Erysipelotrichaceae UCG-004, F0332, and *Leuconostoc*), and chain elongators (*Caproiciproducens*), that cooperate for the caproate production. These microbiomes showed the highest abundance of predicted functional enzymes involved in CE process (e.g., short-chain acyl-CoA dehydrogenase). This study contributed to expand the knowledge on microbial ecology of CE process, providing crucial hints for the performance improvement and engineering of the system up to large-scale applications.

## Data Availability

The datasets presented in this study can be found in online repositories. The names of the repository/repositories and accession number(s) can be found below: https://www.ncbi.nlm.nih.gov/, PRJNA809915 https://www.ncbi.nlm.nih.gov/, PRJNA872912 https://www.ncbi.nlm.nih.gov/, PRJNA872917.

## Abbreviations

CE, chain elongation; EA, electron acceptor; EC, Enzyme Commission; ED, electron donor; FW, food waste; MCFAs, medium chain fatty acids; OLR, organic loading rate; RBO, reverse β-oxidation; SCFAs, short chain fatty acids.
