# Supplementary material for: Ecology of food waste chain-elongating microbiome
Source: Front Bioeng Biotechnol. 2023 Apr 11;11:1157243. doi: 10.3389/fbioe.2023.1157243 (PMC10126515; doi:10.3389/fbioe.2023.1157243)
Supplement: Supplementary file 1 [file DataSheet1.docx]

*Supplementary material*

**Ecology of food waste chain-elongating microbiome**

Simona Crognale^1^*, Alessio Massimi^1^, Michela Sbicego^1^, Camilla Maria Braguglia^1^, Agata Gallipoli^1^, Giulio Gazzola^1^, Andrea Gianico^1^, Barbara Tonanzi^1^, Francesca Di Pippo^1^, Simona Rossetti^1^.

^1^Water Research Institute, National Research Council of Italy, CNR-IRSA, Area della Ricerca RM1, Via Salaria km 29.300, 00015 Monterotondo, Roma, Italy.

*corresponding author:

Dr. Simona Crognale

Water Research Institute (IRSA) - National Research Council of Italy (CNR)

Via Salaria km 29.300, Monterotondo, Rome, 00015, Italy

simona.crognale@irsa.cnr.it

Table S1. Main parameters monitored and quantified during reactors operation. The maximum process efficiency was calculated as organic matter (in terms of total COD) conversion into VFAs (from acetic to caproic).

| **Reactor #** | **Feeding strategy** | **OLR (gCOD/Ld)** | **day** | **Acetate (mg/L)** | **Propionate (mg/L)** | **Isobutyrate (mg/L)** | **Butyrate (mg/L)** | **Isovalerate (mg/L)** | **Valerate (mg/L)** | **Caproate (mg/L)** | **Caproate (%VFA)** | **Ethanol (mg/L)** | **Lactate (mg/L)** | **Efficiency *(gVFA/gCODfed)** |
| --- | --- | --- | --- | --- | --- | --- | --- | --- | --- | --- | --- | --- | --- | --- |
| **1)** | Continuous | 5 | 12 | 2466 | 2869 | 62 | 688 | 0 | 414 | 0 | 0 | 0 | 0 | 0.32 |
|  |  |  | 19 | 2172 | 977 | 69 | 2383 | 43 | 1809 | 240 | 3 | 0 | 0 |  |
|  |  |  | 26 | 2188 | 918 | 88 | 3081 | 64 | 1981 | 504 | 6 | 479 | 0 |  |
|  |  |  | 33 | 3201 | 1059 | 67 | 1796 | 63 | 1742 | 1567 | 17 | 576 | 1032 |  |
|  |  |  | 39 | 3788 | 1602 | 83 | 1795 | 59 | 1923 | 1461 | 14 | 311 | 305 |  |
|  |  |  | 46 | 3582 | 1849 | 90 | 1364 | 60 | 1625 | 953 | 10 | 386 | 1275 |  |
| **2)** | Continuous | 15 | 12 | 5933 | 294 | 0 | 2537 | 43 | 1500 | 4492 | 30 | 553 | 7849 | 0.18 |
|  |  |  | 19 | 7955 | 189 | 0 | 1650 | 0 | 471 | 2237 | 18 | 1143 | 17188 |  |
|  |  |  | 26 | 7173 | 420 | 0 | 2080 | 0 | 381 | 2948 | 23 | 1036 | 18474 |  |
|  |  |  | 32 | 6132 | 430 | 0 | 3031 | 0 | 993 | 4590 | 30 | 1188 | 8501 |  |
| **3)** | Discontinuous | 15 | 11 | 7455 | 1144 | 182 | 4268 | 93 | 2397 | 3454 | 18 | 1132 | 6 | 0.32 |
|  |  |  | 18 | 8780 | 994 | 193 | 3985 | 0 | 1980 | 3530 | 18 | 2252 | 4486 |  |
|  |  |  | 24 | 5955 | 1194 | 0 | 2743 | 0 | 1366 | 4445 | 28 | 2547 | 6031 |  |
| **4)** | Discontinuous | 15 | 11 | 5077 | 3483 | 87 | 6055 | 129 | 133 | 166 | 1 | 102 | 12 | 0.37 |
|  |  |  | 18 | 6584 | 3190 | 150 | 9316 | 219 | 281 | 803 | 4 | 266 | 84 |  |
|  |  |  | 25 | 7408 | 1805 | 113 | 7721 | 141 | 279 | 2033 | 10 | 155 | 0 |  |
|  |  |  | 32 | 11641 | 4749 | 867 | 9412 | 264 | 849 | 3992 | 13 | 323 | 87 |  |
|  |  |  | 39 | 9532 | 2469 | 769 | 5776 | 166 | 1130 | 5912 | 23 | 332 | 98 |  |
|  |  |  | 46 | 7623 | 1783 | 679 | 3826 | 134 | 1271 | 6894 | 31 | 412 | 48 |  |
|  |  |  | 53 | 5041 | 1151 | 669 | 7171 | 127 | 2098 | 4940 | 23 | 1084 | 44 |  |
| **5)** | Discontinuous | 20 | 11 | 3495 | 453 | 101 | 1171 | 78 | 487 | 2891 | 33 | 3255 | 19327 | 0.23 |
|  |  |  | 18 | 4532 | 1703 | 58 | 796 | 0 | 287 | 1625 | 18 | 3757 | 23270 |  |
|  |  |  | 25 | 3791 | 1987 | 0 | 871 | 0 | 251 | 724 | 10 | 3391 | 22495 |  |
|  |  |  | 32 | 5064 | 2473 | 0 | 1304 | 0 | 397 | 511 | 5 | 4188 | 25679 |  |
|  |  |  | 39 | 4021 | 1792 | 0 | 1275 | 0 | 383 | 297 | 4 | 4096 | 25760 |  |
|  |  |  | 46 | 1867 | 2340 | 0 | 12007 | 0 | 951 | 778 | 4 | 4602 | 11571 |  |
|  |  |  | 53 | 1470 | 1592 | 0 | 9364 | 0 | 1032 | 2595 | 16 | 3188 | 5107 |  |
|  |  |  | 58 | 2553 | 1515 | 0 | 9250 | 0 | 1832 | 8100 | 35 | 4205 | 387 |  |
|  |  |  | 67 | 1495 | 1010 | 0 | 6838 | 0 | 1440 | 6480 | 38 | 3902 | 120 |  |
| **6)** | Discontinuous | 20 | 11 | 6068 | 2546 | 380 | 9297 | 103 | 3952 | 3051 | 12 | 3097 | 3 | 0.26 |
|  |  |  | 18 | 4279 | 2012 | 331 | 10687 | 156 | 6333 | 2480 | 9 | 3055 | 32 |  |
|  |  |  | 25 | 4993 | 1418 | 375 | 5004 | 88 | 3607 | 4662 | 23 | 2764 | 8966 |  |
|  |  |  | 32 | 4928 | 709 | 95 | 2394 | 0 | 1707 | 2232 | 19 | 3566 | 25489 |  |


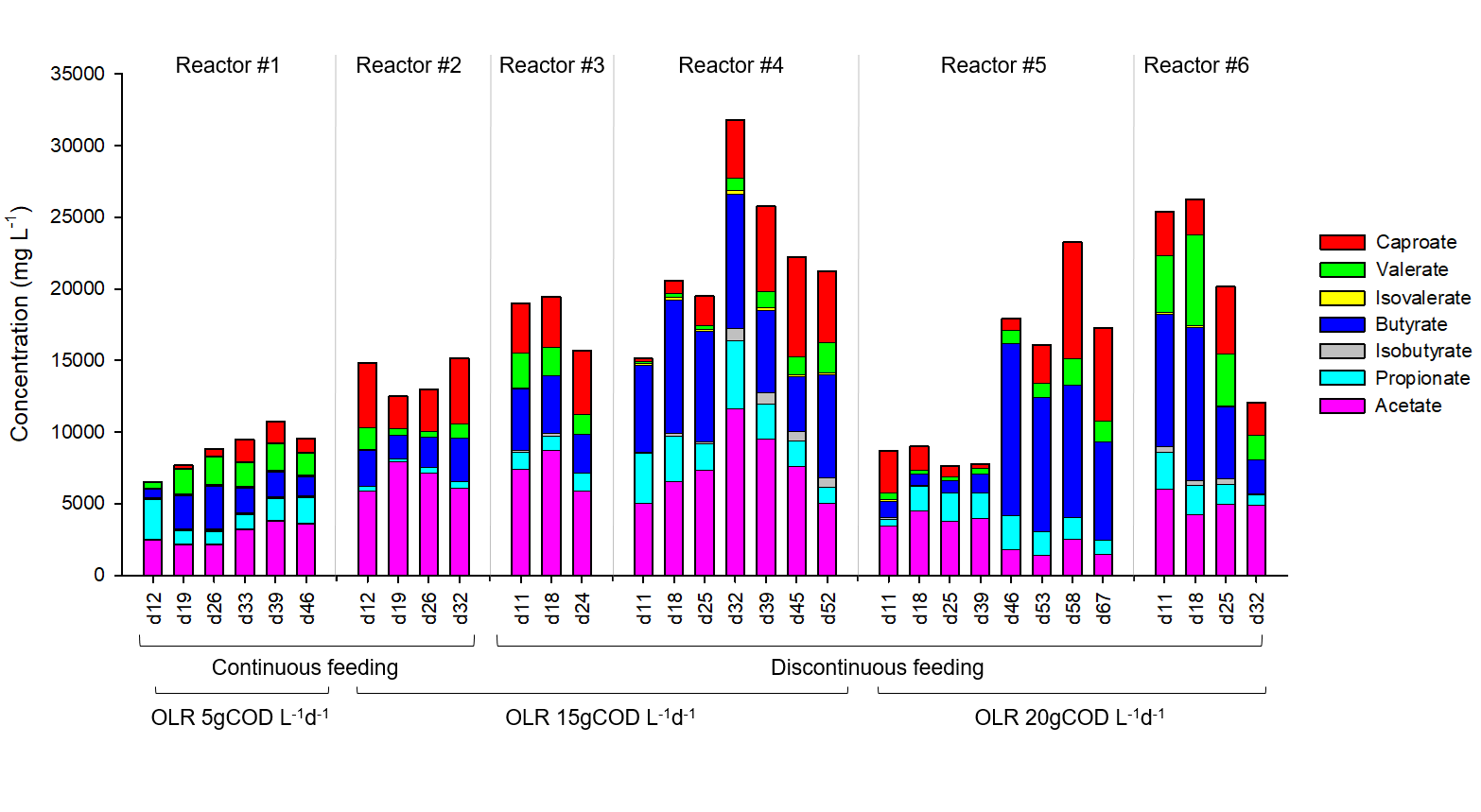


Fig. S1 Daily concentration (mg L^-1^) profile of VFAs during all tests.


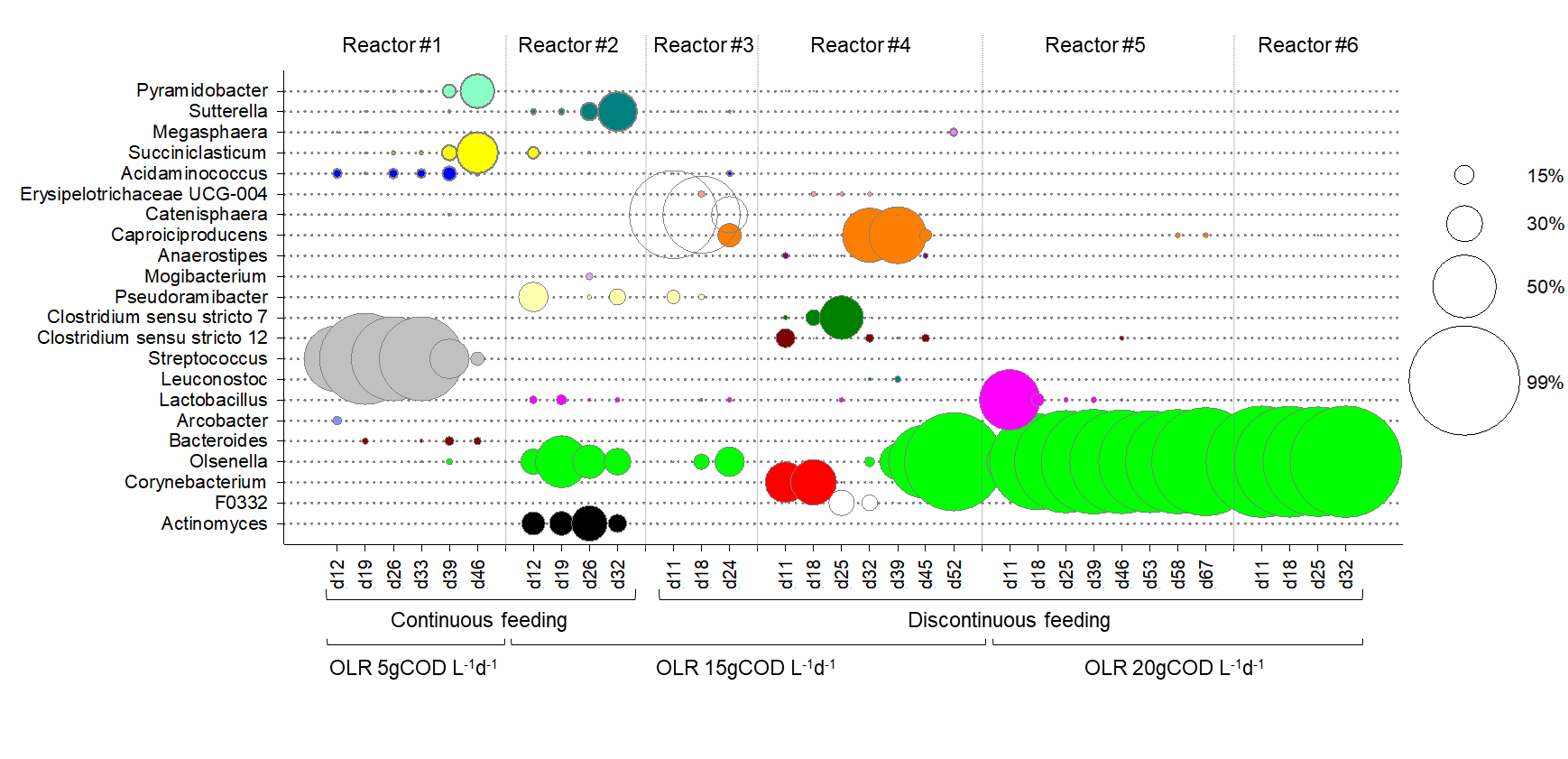


Fig. S2 Bubble plot depicting the relative abundance (as % of total reads) of the main genera (≥5% in at least one sample) in the biomass reactors at different OLRs and feeding strategy.


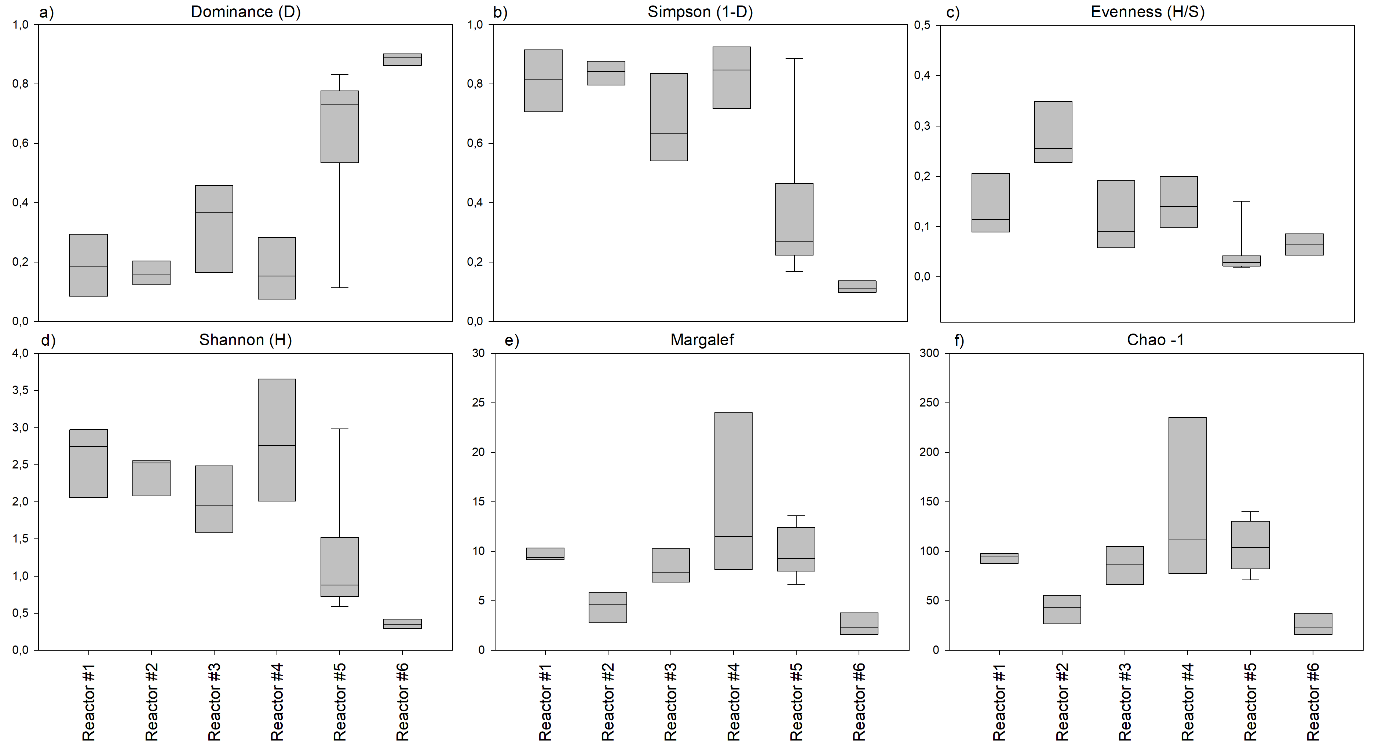


Fig. S3 Boxplot of Alpha-diversity indices: a) Dominance (D), b) Simpson (1-D), c) Evenness (H/S), d) Shannon (H), Margalef, and Chao-1. These indices reflect the measure of diversity considering the number of taxa present (at ASVs level), as well as the relative abundance of each taxon. Boxes represent the interquartile range (IQR) between the first and third quartiles (25th and 75th percentiles, respectively), and the horizontal line inside the box defines the median.
